# The fundamental disturbance (Grundstörung) of schizophrenia (Part 1): Acquiring a potent endophenotype, exploratory eye movement

**DOI:** 10.1002/pcn5.70234

**Published:** 2025-11-18

**Authors:** Takuya Kojima, Eisuke Matsushima

**Affiliations:** ^1^ Medical Corporation Hojinkai Ohmiya Kosei Hospital Saitama‐ken Japan; ^2^ Shirogane‐Takanawa Clinic Tokyo Japan

**Keywords:** endophenotype, exploratory eye movements, responsive search score, schizophrenia, vulnerable predispositions

## Abstract

It is important to identify the common fundamental disturbance (Grundstörung) in schizophrenia and to elucidate its real nature. A fundamental disturbance refers to a primary disturbance underlying the pathology of schizophrenia and is defined as something that can explain various pathological conditions. However, these aspects have not yet been determined. We have investigated them using exploratory eye movements. In this article, we describe our discovery of a potent endophenotype (Part I), while our elucidation of the fundamental disturbance will be presented in the next report (Part II). Exploratory eye movements are movements of the eye fixation ‐ point during free observation of objects. We found that these movements reflect an “attitude to get involved voluntarily and actively in the environment,” in other words, “subjective activity.” We also calculated the Responsive Search Score from exploratory eye movements recorded under conditions of interpersonal interaction and social recognition. This score was characteristically lower in patients with schizophrenia than in healthy individuals and patients with other psychiatric disorders. In addition, it reflected genetic predisposition to schizophrenia and was highly correlated with negative symptoms and the performance intelligence quotient on the Wechsler Intelligence Scale. Furthermore, the characteristics of the score with respect to genetic predisposition met all the criteria for an endophenotype. The Responsive Search Score appeared to be a potent marker for schizophrenia. Hence, we will elucidate the fundamental disturbance of schizophrenia by using this marker.

## INTRODUCTION

International diagnostic criteria, such as the Diagnostic and Statistical Manual of Mental Disorders (DSM)^1^ and the International Classification of Diseases (ICD),^2^ have been widely used for the diagnosis of schizophrenia. These diagnostic criteria are based on psychiatric symptoms, focusing on facilitating consistent diagnosis. Although the reliability of the criteria has been maintained, their validity, or their nature, has not been verified. Various approaches have been applied to diagnose this disorder, including psychopathological, physiological, biochemical/pharmacological, and genetic methods. The psychopathological methods have enhanced the pathological understanding but raised issues that need to be clarified by the biological methods. Through the biochemical/pharmacological methods, antipsychotic agents have been developed and clinically applied. These agents have greatly contributed to the treatment of hallucinations and delusions in the acute phase and have also been effective as maintenance therapy for prevention of relapse. Based on the mechanisms of action of these agents, hypotheses have been generated, including the dopamine excess hypothesis,^3^ the N‐methyl‐D‐aspartate (NMDA) receptor hypofunction hypothesis,^4^ and the gamma‐aminobutyric acid (GABA) neuron hypothesis.^5^ These hypotheses can account for some, but not all of the phenomena caused by schizophrenia. With the advances in molecular genetic methods, genetic studies have revealed many related genes. However, the penetrance of each gene is so low that the genetic architecture may be polygenic,^6^ and new approaches are being explored. Recent research on brain image analysis, a physiological method, has advanced the understanding of schizophrenia.^7^, ^8^, ^9^ Schizophrenia is a brain development disorder, including disorders of central nervous circuits such as the frontal lobe, temporal lobe, and thalamus. These disorders are also associated with genetic findings.^7^ In addition, brain image analysis has been combined with analytical technology using computers (computational psychiatry), leading to the development of a new field. The studies in this new field have shown that impairment of the predictive function, which is important for humans, is associated with hallucinations and delusions characteristic of schizophrenia.^10^, ^11^ Meanwhile, physiological studies conducted in a more clinical setting have focused on eye movement as an indicator of the characteristics of schizophrenia. While various eye movements are used to identify abnormalities in schizophrenia,^12^ recent studies have demonstrated that, among three types of typical eye movement tests, examination of eye movements under free conditions (free viewing) is the most effective method with which to differentiate schizophrenia and non‐schizophrenia.^13^, ^14^ Exploratory eye movements, which we have been studying, are the exact eye movements which occur under free conditions. Thus, we attempted to elucidate the fundamental disturbance (Grundstörung) and real nature of schizophrenia by using exploratory eye movements. Since our findings are extensive, they are reported separately in Parts I and II. In this article, we discuss whether exploratory eye movements are the endophenotype of schizophrenia. Although this study has previously been reported in a summarized form,^15^ the present paper significantly differs from and is clearly superior to the earlier report in both quantity and quality.

### Fundamental disturbance, subjective activity, and endophenotype

Schizophrenia is primarily diagnosed when symptoms such as auditory hallucinations, delusion, incoherent speech, catatonic symptoms, and negative symptoms persist for one month or longer.^1^ However, these symptoms vary among patients, and no symptoms have been identified as fundamental disturbance common to all patients with schizophrenia. Although studies show that cognitive dysfunction is a predictor for the prognosis of schizophrenia, Schaefer et al.^16^ report that various cognitive functions were equally impaired in patients with schizophrenia and that there was no impaired cognitive function specific to schizophrenia.

Conversely, Bleuler^17^ maintained the view that the dissociation of thinking was a primary disturbance and also considered the weakening of will‐power, emotional stiffness, flattening, and ambivalence, although to a lesser extent, to be the primary disturbance. Delusions, hallucinations, and catatonic symptoms are regarded as secondary or accessory. Bleuler was searching for a fundamental disturbance, hoping that the symptoms could then be explained as the reaction of the patients’ mind to this disturbance.^18^

In the 1960s, fundamental disturbances of schizophrenia were actively discussed from the viewpoint of clinical psychiatry. A fundamental disturbance (Grundstörung) refers to a primary disturbance underlying the pathology of schizophrenia and is defined as something that can explain various pathological conditions.^19^ This discussion presented disorders caused by facing reality,^20^ disorders of interpersonal behaviors,^21^ praecox feeling,^22^ etc. Although these disorders indicated problems with subjectivity and activity, they have not been incorporated into existing diagnostic criteria. However, recent advances in imaging analysis and neuroscience have revealed that sensory input associated with a movement occurs before the start of the movement, thereby leading to the prediction of the movement.^10^ Hence, humans are actively involved in their environment, rather than being passively involved.

In biological research, biomarkers are used as characteristic indicators of psychiatric disorders. There are state markers and trait markers, the former reflecting the state, and the latter reflecting predisposition. Recently, the concept of endophenotypes has been proposed as indicators that represent both clinical phenotypes and genetic predispositions.^23^ It is thought that these markers will be of great help in elucidating the fundamental disturbance. Therefore, we clarified whether the markers considered in this paper are characteristic of schizophrenia, and at the same time examined whether they strictly meet the criteria for endophenotypes and whether they represent the subjective activity. Finally, we clarified whether these markers could be an important tool for studying the fundamental disturbance.

### Characteristics of movement of eye fixation ‐ point (eye movements)

Visual perception had been considered to occur only after input of information. However, Neisser^24^ proposed that an active process (formula) composed of prediction, expectation, etc. for the target is performed before visual perception. According to this formula, the eye fixation‐point moves to search for the target, and the formula is corrected based on the obtained information. Subsequently, the eye fixation‐point is reoriented to search for and obtain new information, and the formula is corrected again. Neisser stated that perception occurs through such cycles. In this formula, eye movement plays a role in exploratory behavior. Prediction and exploration are so closely associated that they can be unified. Thus, examination of eye movement permits examination of the patterns of prediction, which are active attitudes and behaviors related to the target, known as subjectivity and activity. In terms of physiology and anatomy, clear vision of a target requires its image to be focused on the fovea, a small area where photoreceptors are packed on the retina. Hence, the eyes move. Eye movement is associated with the will and intention of a subject to see the target. Thus, the movement of eye fixation or eye movement is an extremely characteristic indicator that reflects the active attitudes of the subject toward the environment (subjectivity/activity).

In this study, we clarify how our studies on eye movement^25^, ^26^ led us to conclude that disturbances of subjective activity are the fundamental disturbance of schizophrenia (elucidated in Part 2). Here, “activity” refers to attitudes and behaviors to purposefully and actively deal with environmental and stimuli. In our previous studies, this feature was defined as a disturbance of subjectivity ^27^), but the term “subjectivity” is ambiguous. Therefore, defining this feature as “subjective activity” focuses on the basic term “activity” while the term “subjectivity” is used to indicate “voluntary involvement.” In this article, the disturbance of activity refers to the “disturbance of subjective activity.”

### Changes in eye fixation‐ point during free‐hand and retention tasks

Using the hand‐made eye‐tracking recorder developed at the commencement of their study, Moriya et al.^28^ recorded movements of eye fixation in patients with chronic schizophrenia and healthy individuals who were shown horizontal S‐shaped geometric figures and illustrations for a psychological test (Thematic Apperception Test [TAT] illustrations). They reported that movements of eye fixation are limited and not expanded in patients with schizophrenia. In the subsequent studies by Moriya^29^ and Moriya et al.,^30^ they examined the process of visual perception in patients with schizophrenia and their parents with commercially available eye‐tracking recorders. After presenting a horizontal S‐shaped figure and a concrete illustration (depicting a prince and a princess playing in front of a castle), they initially asked participants to observe the figures freely and examined movements of eye fixation. Subsequently, participants received the instruction, “Please observe the pictures carefully because you will be asked to draw them later,” and movements of eye fixation were evaluated. In addition to factor indicators of eye fixation‐point, such as eye fixation time, number of eye fixation and total eye scanning length, Moriya et al. evaluated the extent to which eye fixation‐point remained to the parts necessary for recognizing the figures, and the degree of effective exploration was calculated. Consequently, in healthy individuals, eye fixation time was mostly short, ranging from 0.25 to 0.4 s with little variation (variability), and their eye fixation‐point moved frequently (high number of eye fixation) in a wide area (long total eye scanning length). They also looked at the parts important for recognizing figures (high degree of effective exploration). However, in patients with schizophrenia, eye fixation time was mostly long with large variation (large variability), and their eye fixation‐point moved less frequently (low number of eye fixation) in a small area (short total eye scanning length). The degree of effective exploration was low. As for the movement of eye fixation of the parents of patients with schizophrenia, these indicators showed intermediate values between those of the patients with schizophrenia and healthy individuals. Among the participants, patients with schizophrenia most poorly reproduced the figures. When reactions were compared before and after the second instruction, healthy individuals displayed shorter eye fixation time, higher number of eye fixation and longer total eye scanning length, and carefully observed every part of the picture. However, patients with schizophrenia showed no apparent changes in their movement of eye fixation after the second instruction, with their parents only displaying changes in number of eye fixation. These results imply that patients with schizophrenia and their families are less likely to actively react to their external environment and its changes. This supports the weak prediction (formula) mentioned by Neisser and appears to be a disorder of “subjective activity.” Moreover, the parents of the patients displayed abnormal values that were intermediate to those of the healthy individuals and patients with schizophrenia. These results suggest that these indicators reflect the predispositions to schizophrenia. Subsequently, there have been many reports of studies in which eye movements were recorded while the participants were freely observing the pictures.^31^, ^32^, ^33^, ^34^, ^35^ As seen in the studies conducted by Moriya and us, short eye scanning length, and low number of eye fixation, etc. were observed in patients with schizophrenia, compared with healthy individuals.

### Changes in the eye fixation‐point during recognition and reminder tasks

**Figure 1:**
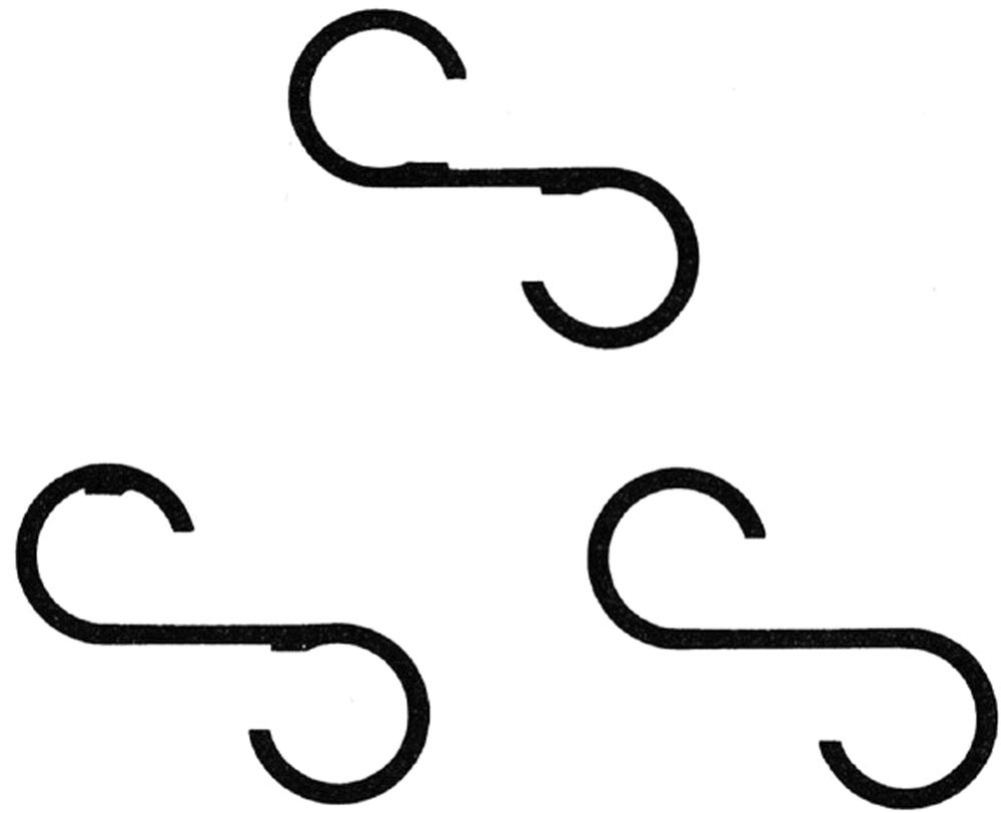
The three *S*‐shaped figures used. The two figures at the bottom are slightly different from the target figure shown at the top.^36^

In patients with schizophrenia, their eye fixation‐point moved less frequently within a small area, and their reproduced figures were poor. These results suggest that the patients could not reproduce the figure well because they did not observe it carefully. Thus, we believed that we needed to help the participants observe the figure carefully to enable accurate reproduction. Participants received the instruction, “Please compare the picture that you first saw and the pictures that we will show and decide whether they are identical or different.” Subsequently, we displayed a total of three figures, consisting of two figures partially differing from the target figure and one figure identical to the target figure (Figure 1). Each figure was displayed for 15 s in the order of a different figure, the identical figure, and a different figure (recognition task). Immediately after the figures were shown, participants were asked to respond whether each figure was identical to or different from the target figure and what the differences between them were. Finally, participants were asked a reminder question, “Are there any other differences?” We recorded eye movement for 5 s from the moment when we pronounced the first word of the reminder question (reminder task).^36^, ^37^

**Figure 2:**
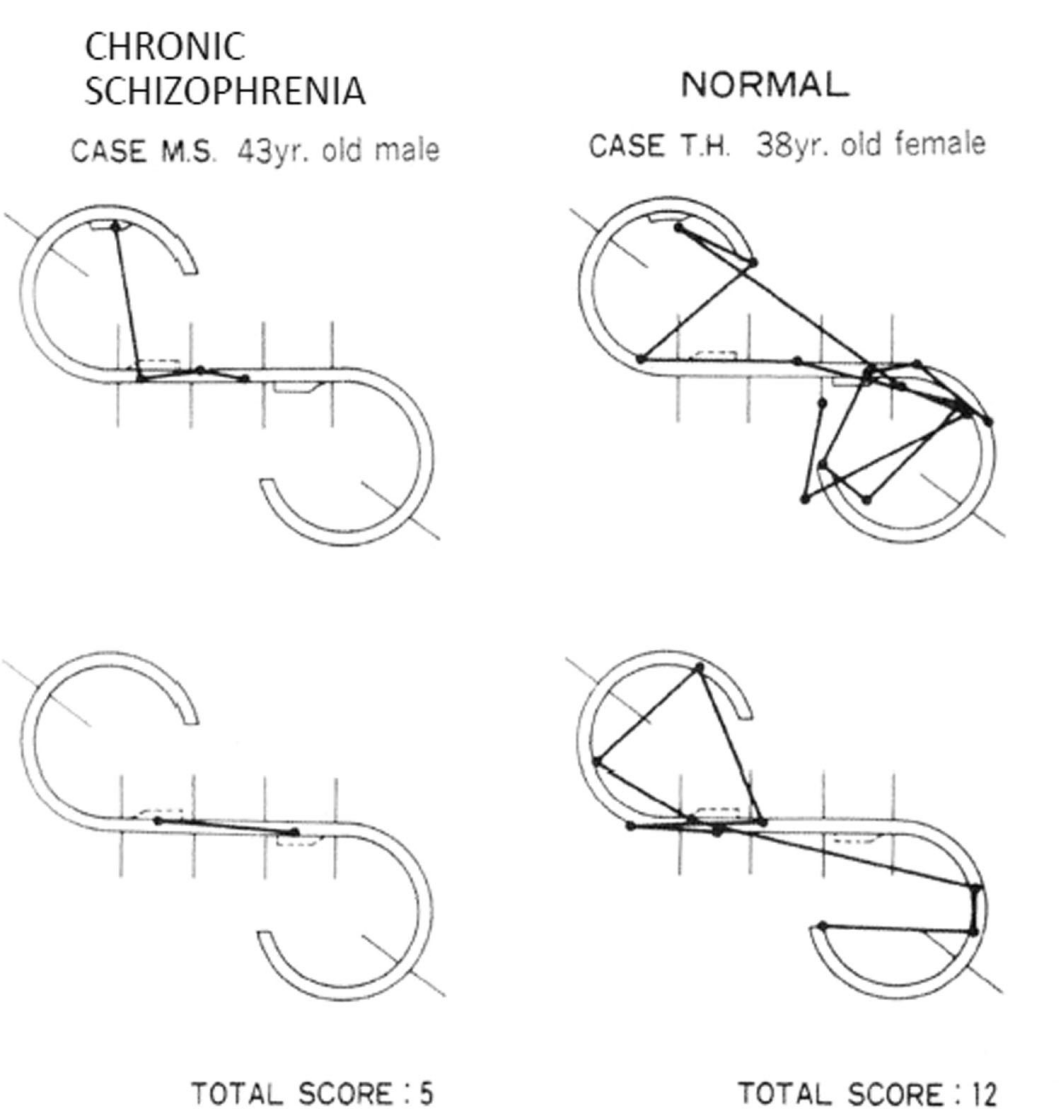
Examples of responsive search scores of a patient with chronic schizophrenia and a normal control. The top and bottom figures are slightly different from the original. The normal control shows frequent eye movements while guessing the intention of the examiner and questioning themselves “Are there still other differences?” Although he answered, “There are no more differences,” his eye fixation‐point actively moved immediately before answering. The number of the sections his eyes focused on was six in the top figure and six in the bottom. The total score is 12. The patient with chronic schizophrenia displays less frequent eye movements before answering, “No other difference.” The total score for the patient with chronic schizophrenia is 5.^36^

In healthy individuals, movements of eye fixation during the reminder task were markedly active and notably differed from the slightly increased number of eye fixation‐point in the patients with schizophrenia. The degree of movement of eye fixation during this task was referred to as the Responsive Search Score (RSS). (Figure 2)

### Determination of the Responsive Search Score during the reminder task^25^, ^26^, ^36^, ^37^, ^38^, ^39^

When the reminder question was asked, healthy individuals guessed the intention of the examiner and questioned themselves, “Are there still other differences?” Although they answered, “There are no more differences,” their eye fixation‐point actively moved immediately before answering. Similarly, patients with schizophrenia answered, “There are no more differences,” but their movement of eye fixation was limited (Figure 2). A close examination of these circumstances suggests a process consisting of guessing the intention of the examiner, reviewing and confirming the answer, and making a decision (delivery of the answer). This is exactly the process performed during interpersonal interaction (social recognition). The RSS, which indicates exploratory eye movement in this situation, reveals maximally enhanced “subjective activity.”

### The Responsive Search Score reflecting the subjective activity: A study using a button pressing task^27^

The button pressing task was assigned to the participants to check if the RSS represented subjective activity. The examiner presented a total of three figures, consisting of the target figure and two partially differing figures in the order of the different figure, the identical figure, and the different figure. The participants were asked to press the button when they determined whether the presented figure was identical to or differed from the target figure. The movement of eye fixation was recorded during the period until the button was pressed (Search Score at the time of recognition) and the 5 s period after pressing the button (Search Score after recognition). The examiner verbally asked the participants whether the presented figure was different from or the same as the target figure. When they answered that the figures were different, the examiner inquired further about how different the figures were. Furthermore, the examiner asked a reminder question about whether there were any other differences, and the movement of eye fixation was recorded for 5 s from the moment when the first word of the reminder question was pronounced (Responsive Search Score: RSS). The percentage of participants who correctly answered whether the presented figures differed from or were the same as the target figure did not significantly differ between the patients with schizophrenia and healthy individuals. From the records of eye movement, we determined how many of the seven sections the eye fixation‐point remained during the period until the button was pressed in the recognition task. The number of sections was regarded as the Search Score. Among healthy individuals, scores were significantly higher when the figure identical to the target figure was presented than when the two different figures were presented. Conversely, patients with schizophrenia showed no difference between their scores for identical and different figures (Search Score at the time of recognition). Regarding the eye movement recorded for 5 s after pressing the button, healthy individuals moved their eye‐fixation point to all the figures more actively and had higher scores than the patients with schizophrenia (Search Score after recognition, which was calculated in the same manner as the Search Score at the time of recognition). When the reminder question was stated, the healthy individuals moved their eye fixation‐point far more actively and had higher scores than the patients with schizophrenia (RSS). When the healthy individuals were asked whether the presented figure differed from or was the same as the target figure (Search Score at the time of recognition) and shown the identical figure, the formula (state of prediction/preparation) was activated immediately before pressing the button to make them think, “I want to closely examine the figure because it looks similar.” Subsequently, they closely examined whether the figure differed or was the same. After pressing the button (Search Score after recognition), the formula was activated to make them think, “I want to check whether my answer is correct.” Subsequently, they confirmed their decision to press the button. In the reminder task performed under the tense condition of the face‐to‐face interview, the participants instantly reacted to the reminder question and re‐examined the figure in front of them with the intention of saying, “There is no difference.” This suggests the activation of the formula to make the subjects think, “I want to examine the figure in front of me again with regard to my words” (answer). While these three scores were recorded, the participants re‐examined the figures and their decisions immediately before and after they determined their final answers. This indicated an active voluntary action performed toward the target by the participants to obtain the correct answer, or, the subjective and active action of confirmation. As all three scores strongly reflected subjective activity, exploratory eye movement was validated to reflect subjective activity. In addition, the contents of confirmation/re‐examination were (1) confirmation/re‐examination of whether the presented figure differed from or was the same as the target figure, (2) confirmation/re‐examination of the participant's action to press the button, and (3) confirmation/re‐examination of the participant's words in the interview setting. We assumed that as the action of confirmation/re‐examination progresses from (1) to (3), the psychological distance between the confirmation action and self (subjectiveness) is shortened, thereby generating more severe tension and stronger subjective activity. Furthermore, the RSS appeared to require stronger subjective activity with respect to reactions under the condition of social recognition, unlike the other two scores, as described above. When the correlation among the three scores was examined, the RSS correlated with both the Search Score at the time of recognition (*r* = 0.37, *P* < 0.05) and the Search Score after recognition (*r* = 0.36, *P* < 0.05), whereas no correlation was observed between the Search Score at the time of recognition and the Search Score after recognition. When the two scores that were calculated from the same 5 s records were also compared, the RSS (4.09) was higher than the Search Score after recognition (3.09,) (*P* < 0.0001). These findings show that these three scores represent subjective activity and that the RSS is the most potent indicator among them.

### Is a low Responsive Search Score a feature of schizophrenia?

We examined RSSs of patients with schizophrenia,^25^, ^26^, ^36^, ^37^, ^38^, ^39^ mood disorder,^26^, ^37^, ^40^ neurosis,^26^, ^37^, ^40^ frontal lobe disorder,^41^ Parkinson's disease^25^, ^26^ epilepsy,^36^, ^40^ systemic lupus erythematosus (SLE),^42^ or methamphetamine psychosis,^37^, ^43^ healthy individuals, etc. The RSSs of patients with disorders other than schizophrenia and healthy individuals were all significantly higher than those of patients with schizophrenia. No groups of patients with a psychiatric disorder are associated with low RSSs, except for those with schizophrenia. Furthermore, in the World Health Organization multicenter study^44^ conducted at seven institutions in six countries that compared patients with schizophrenia with healthy individuals and patients with depression, the significant differences observed in number of eye fixation and total eye scanning length during the retention task varied among the institutions (Table 1‐1). However, the RSSs were significantly lower among patients with schizophrenia than among patients with depression and healthy individuals at all institutions, with no significant difference between patients with depression and healthy individuals (Table 1‐2). The study demonstrated that the RSS is extremely specific to schizophrenia and is consistently low across races and cultures.

**Table 1‐1:** Number of eye fixation‐point.

WHO center	Schizophrenia (S)	Depression (D)	Control (C)	S vs D	D vs C	S vs C
Beijing	30.6 ± 7.2	34.5 ± 7.2	34.9 ± 5.0			
Casablanca	24.0 ± 9.7	30.6 ± 6.7	33.2 ± 5.3			*
Montreal	28.0 ± 9.0	33.6 ± 7.3	38.4 ± 7.1			*
Munich	27.3 ± 10.1	37.1 ± 5.3	37.0 ± 7.4	*		*
Prague	22.1 ± 6.9	30.4 ± 7.6	35.3 ± 5.9	*	*	*
Sapporo	25.4 ± 6.8	34.1 ± 6.7	33.4 ± 4.6	*		*
Tokyo	33.0 ± 6.1	33.7 ± 8.2	38.8 ± 7.3		*	*
All	27.4 ± 8.6 (Mean ± SD)	33.2 ± 7.4	36.3 ± 6.5	*	*	*

* *p* < 0.05: Multiple comparison for Two‐way ANOVA.

**Table 1‐2:** Responsive Search Score.

WHO center	Schizophrenia (S)	Depression (D)	Control (C)	S vs D	D vs C	S vs C
Beijing	7.4 ± 2.2	10.6 ± 1.4	10.8 ± 1.3	*		*
Casablanca	6.7 ± 1.6	10.3 ± 0.7	10.3 ± 1.4	*		*
Montreal	6.9 ± 1.6	9.9 ± 1.2	9.8 ± 1.3	*		*
Munich	8.2 ± 2.1	10.3 ± 0.9	11.2 ± 1.0	*		*
Prague	7.5 ± 2.2	11.2 ± 1.1	11.1 ± 1.4	*		*
Sapporo	7.2 ± 1.7	10.0 ± 1.4	10.7 ± 1.4	*		*
Tokyo	7.9 ± 1.7	10.6 ± 1.4	10.6 ± 1.9	*		*
All	7.5 ± 1.9 (Mean ± SD)	10.5 ± 1.3	10.8 ± 1.5	*		*

* *p* < 0.05: Multiple comparison for Two‐way ANOVA.^36^

### Associations of the Responsive Search Score with psychiatric symptoms and neuropsychological tests

We examined the associations of the RSS with psychiatric symptoms and neuropsychological tests. Psychiatric symptoms were evaluated with the Brief Psychiatric Rating Test (BPRS),^45^ the Scale for Assessment of Negative Symptom (SANS),^46^ and the Schedule for Affective Disorders and Schizophrenia (SADS).^47^ The three neuropsychological tests examined were the Wechsler Intelligence Scale (WAIS),^48^ the Maze test from the Wechsler Intelligence Scale for Children‐Revised,^49^ and the New Modified Wisconsin Card Sorting Test (WCST)^50^ modified by Kashima et al. The RSS was highly correlated with WAIS performance intelligence quotient (IQ) (correlation coefficient: 0.74), the Block Design test (0.67), and the Object Assembly test (0.76). The RSS was also correlated with the Maze test (0.55). As for psychiatric symptoms, the RSS was correlated with the SANS subscale of affective flattening or blunting (−0.50), avolition‐apathy (−0.64), and attention dysfunction (−0.62), and the BPRS on emotional withdrawal (−0.52), and blunted affect or inappropriate affect (−0.57). By contrast, the RSS was not correlated with WAIS verbal IQ, WCST, or positive symptoms. The RSS correlated with the WAIS performance IQ and maze test, which are thought to represent right hemisphere function, but did not correlate with the WCST or WAIS verbal IQ, which are related to left hemisphere function. This pattern strongly suggests a relationship between the RSS and right hemisphere function.

It was noteworthy that the RSS was highly correlated with negative symptoms and WAIS performance IQ.

### Responsive Search Score reflecting the vulnerable predispositions to schizophrenia

Takahashi et al.^51^ compared RSSs and the number of eye fixations in responsive search (NEFRS) between patients with schizophrenia, their healthy siblings, and age‐matched healthy individuals. The RSSs and NEFRS were significantly lower among patients with schizophrenia and their siblings than in healthy individuals. In addition, the RSSs and NEFRS were significantly lower in patients with schizophrenia than in their siblings. Thus, the values of healthy siblings were between those of the patients with schizophrenia and healthy individuals. Takahashi et al.^52^ performed a linkage analysis of schizophrenia with NEFRS as an indicator and found a linkage to the chromosome region 22q11.2‐q12.1. The chromosome region 22q11 is among the regions attracting the greatest attention in schizophrenia, and patients with 22q11.2 deletion syndrome are frequently diagnosed with schizophrenia.

Yara et al.^53^ reported that the RSSs of patients with schizophrenia and patients with mood disorder who had first‐degree relatives with schizophrenia were significantly lower than the RSSs of patients with mood disorder who had a family history of mood disorder, patients with mood disorder who did not have a family history of psychiatric disorder, and healthy individuals, and that there was no significant difference between the two groups of patients with mood disorder who did not have a family history of schizophrenia and healthy individuals. Based on these findings, Yara et al. identified the predisposition to schizophrenia among patients with mood disorder. Kojima and Matsushima^54^ examined the RSS of monozygotic twins with schizophrenia and healthy controls, reporting a high correlation coefficient (0.83) in monozygotic twins. They also studied multiplex families with schizophrenia and compared patient RSS across pedigrees with one, two, or three affected first‐degree relatives and found that patients with three affected first‐degree relatives had the lowest scores. These results indicate that the RSS reflects vulnerable predispositions to schizophrenia.

### The effects of antipsychotics on Responsive Search Score

In a study^39^ conducted by Kojima T. et al., there was no significant difference in eye movements between 15 neuroleptic‐treated and 14 medication free patients with schizophrenia. The RSS of 10 patients with schizophrenia were examined twice (on medication and off medication). There was no significant difference in RSS between the on‐and‐off‐medication states. Furthermore, relatives of patients with schizophrenia^30^, ^51^, ^53^ showed lower RSS values than those for healthy controls and higher values than those for patients with schizophrenia, reflecting a predisposition to schizophrenia. Taking these findings together, we believe that antipsychotic drugs have little effect on RSS and exploratory eye movements.

### Responsive Search Score representing the endophenotype of schizophrenia

The endophenotype is a neurobiological phenotype that shows features of both a clinically diagnosed psychiatric disorder and genes. Linkage and association analyses based solely on clinical diagnosis often result in inconsistencies among researchers. This suggests that differences in the phenotypes of psychiatric disorders with common genetic predisposition affect analysis outcomes. Using the endophenotype, instead of the psychiatric disorder, allows researchers to identify a more genetically homogeneous group of patients, which is advantageous for genetic studies.^23^, ^55^, ^56^

In addition, when it comes to identifying the underlying disorders and primary pathology of schizophrenia, the endophenotype, with its distinctive features, is the most appropriate indicator available. In this study, we investigated whether the RSS would meet the criteria for an endophenotype.

The RSS meets all of the six criteria reported for the endophenotype (intermediate phenotype) of schizophrenia by Gershon ES and Goldin LR,^23^ Meyer–Lindenberg and Weinberger,^57^ Hashimoto^58^: (1) being heritable,^29^, ^51^, ^52^, ^53^, ^54^ (2) being quantitatively measurable,^36^, ^37^, ^38^ (3) being related to the psychiatric disorder and its symptom in isolated cases^31^, ^32^, ^33^ (4) being stable over a long period of time^59^ (5) showing the development of schizophrenia even among individuals without psychiatric disorder within a family with a history of psychiatric disorder,^29^, ^51^, ^53^, ^54^ and, (6) being more strongly associated among individuals with psychiatric disorder than among those without psychiatric disorder within a family with a history of psychiatric disorder.^29^, ^51^, ^53^, ^54^ This indicator can represent the endophenotype of schizophrenia. Notably, this indicator is strongly associated with negative symptoms and cognitive function (WAIS Performance IQ). Particularly, negative symptoms are important because they are observed throughout the acute and chronic phases of schizophrenia and are regarded as a key indicator of fundamental disturbance by Kraepelin^60^ and Bleuler,^17^ Additionally, Foussias and Remington^61^ reported that the lack of motivation (i.e., avolition) is the core of negative symptoms. Thus, negative symptoms and the disorders of subjective activity indicated by the RSS are equivalent in terms of directionality. Among neurophysiological indicators, including brain imaging, which are considered to be candidates for the endophenotype of schizophrenia, none have been reported to meet all of the criteria described above or to be clearly correlated with clinical symptoms, particularly negative symptoms^62^, ^63^, ^64^, ^65^, ^66^. For this reason, we would like to elucidate the primary pathology and fundamental disturbance of schizophrenia using the RSS, which clearly and characteristically represents the endophenotype.

## CONCLUSION

We studied schizophrenia using eye movement during free observation of the target, referred to as exploratory eye movement and revealed that this movement reflects subjective activity. Subsequently, we measured exploratory eye movement under the conditions of interpersonal interactions and social recognition as the RSS. This indicator was found to be specific to schizophrenia, reflected genetic predispositions to the disorder, and represented the endophenotype strongly associated with negative symptoms.

No other indicators reflect the features of the endophenotype of schizophrenia as clearly as the RSS.

## AUTHOR CONTRIBUTIONS

Kojima conceptualized this article and prepared the first draft. Discussions with Matsushima led to multiple revisions till the draft was finalized. Matsushima prepared the figures and tables, organized the references, and performed other tasks. This article is a product of cooperation among many researchers over a long period of time. We would like to express our appreciation by listing their names separately.

## CONFLICT OF INTEREST STATEMENT

The authors declare no conflicts of interest.

## ETHICS APPROVAL STATEMENT

This study was conducted after the participants received sufficient explanation of the tests and provided written informed consent. The study was also conducted with approvals from the ethics committees of all participating institutions.

## PATIENT CONSENT STATEMENT

N/A.

## CLINICAL TRIAL REGISTRATION

N/A.

## Data Availability

The data that support the findings of this study are available from the corresponding author upon reasonable request.
